# Effect of High Hydrostatic Pressure on Stress-Related *dnaK*, *hrcA*, and *ctsR* Expression Patterns in Selected *Lactobacilli* Strains

**DOI:** 10.3390/genes12111720

**Published:** 2021-10-28

**Authors:** Joanna Bucka-Kolendo, Edyta Juszczuk-Kubiak, Barbara Sokołowska

**Affiliations:** 1 Department of Microbiology, Culture Collection of Industrial Microorganisms-Microbiological Resource Center, Prof. Wacław Dąbrowski Institute of Agricultural and Food Biotechnology—State Research Institute, 02-532 Warsaw, Poland; 2 Department of Microbiology, Laboratory of Biotechnology and Molecular Engineering, Prof. Wacław Dąbrowski Institute of Agriculture and Food Biotechnology—State Research Institute, 02-532 Warsaw, Poland; edyta.juszczuk-kubiak@ibprs.pl; 3 Department of Microbiology, Prof. Wacław Dąbrowski Institute of Agricultural and Food Biotechnology—State Research Institute, 02-532 Warsaw, Poland; barbara.sokolowska@ibprs.pl; 4 Institute of High Pressure Physics, Polish Academy of Sciences, 01-142 Warsaw, Poland

**Keywords:** lactic acid bacteria (LAB), high hydrostatic pressure (HHP), stress-related genes, gene expression

## Abstract

Lactic acid bacteria (LAB) in the natural environment meet multiple stressors such as pH and temperature variations, increased nutrition and metabolite concentrations, harmful chemicals, acidic/oxidative conditions, osmotic pressure, and starvation. However, LAB strains are not subjected to high hydrostatic pressure (HHP) which currently is the most common non-thermal decontamination technology in the food industry. In this context, the LAB response to HHP is more difficult to identify compared to other stress-induced responses, and *dnaK*, *ctsR*, and *hrcA* can serve as essential regulators in this reaction. In the present study, the expression level of *dnaK*, *ctsR*, and *hrcA* mRNAs in 15 LAB strains after the HHP (300 MPa/5′) exposure was evaluated. As a result, the HHP-treatment affected the up-regulation of *dnaK*, *ctsR*, and *hrcA* in *L. backii* KKP 3565, *L. backii* KKP 3566, *L. rhamnosus* KKP 3570, *L. brevis* KKP 3575 strains, whereas, in *L. plantarum* KKP 3569, *L. rhamnosus* KKP 3571, *L. brevis* KKP 3573 all genes were lower expressed. The relative expression level of the *dnaK*, *ctsR*, and *hrcA* either before or after the pressure treatment for *L. brevis* DSM 6235, *L. rhamnosus* KKP 3572, *L. brevis* KKP 3574, *L. brevis* KKP 3576, *L. rossiae* KKP 3577, *L. curvatus* KKP 3578 strains were undetectable. Significant differences in the expression levels were observed, between the control and the HHP treatment strains for *dnaK* in *L. backii* KKP 3565, *L. backii* KKP 3566, *L. plantarum* KKP 3569, *L. rhamnosus* KKP 3570, *L. rhamnosus* KKP 3571, *ctsR* in, *L. backii* KKP 3565, *L. rhamnosus* KKP 3570, *L. rhamnosus* KKP 3571, and *hrcA* in *L. plantarum* KKP 3569, *L. rhamnosus* KKP 3571. Overall, the studied genes, *dnaK*, *ctsR*, and *hrcA* can be useful markers to indicate the LAB cellular response to HHP. These molecular parameters can help to optimize the desirable LAB growing conditions in industrial processes and to understand the complexity of the stress-related mechanism.

## 1. Introduction

One of the biggest challenges is the clarification of the structure, functions, and dynamics of bacteria in their natural environments, as well as when subjected to different types of stress. Over the past decade, numerous technologies have been developed to investigate the association between bacteria and their reaction to changes in their niches. LAB are desired in many food products where they can provide positive sensory and nutritive features, however in products like juices, beverages, and beer they can cause spoilage and waste. [1]. The stress response is a protective mechanism of bacterial survival and adaptation to harsh environmental conditions [2]. Exposure to a rigorous manufacturing process allows LAB to adjust to growing in or surviving in a hostile environment. The ability to cope with a wide range of environmental stress factors (changes in pH and temperature, a variety of nutrition and metabolite concentration, presence of toxic chemicals, acidic or oxidative conditions, osmotic pressure, and starvation), induce changes or repression of their stress response mechanisms [1,3]. This response is universal as it can be observed in studied bacterial species, and it consists of a set of well-coordinated processes, mainly involving the regulation of the production of various heat-shock proteins. These mechanisms include the activation of proteins responsible for protecting the bacterial cells against stress [2,4]. 

Among the different stress factors that can affect gene expression levels is high hydrostatic pressure (HHP). HHP is a food and beverage products preservation method that will not affect molecules like vitamins, amino acids, or flavor compounds but at the same time reduce the number of microbial counts in the product. LAB in their natural environment does not show a response to HHP, as those bacteria are not normally exposed to this stress factor [1]. It has been reported [5,6] that LAB belongs to a group of bacteria that are resistant to HHP, and because of that, the evaluation of stress response mechanisms to HHP are valuable tools to assess the shelf life of products preserved with this method. However, the LAB response to HHP is more difficult to identify in comparison to other stress factors [3], as their ability to react to HHP is expected to come from the cross-protection system [1]. Where cross-protection in LAB has been described as the exposure to a single stress factor that is commonly associated with cross-tolerance to other stressors like acid, heat, etc. [7]. Studies indicate that the molecular mechanisms of the adaptive response to stress can be species-specific [8], or even strain-specific [9], and it is considered that some phenotypes depend on the level of the expression of genes rather than their presence or absence. Thus, further studies are needed to specify how many are species/strain-specific and how/if the specificity correlates with stress toleration in *Lactobacillus* strains. Differential expression of the genes involved in stress response can result in changes in the phenotypic level [9,10,11]. Although the genes that are correlated with stress response in LAB are highly conservative, [10] including the high conserved class I regulon [1,12], the mechanisms of stress responses are not conserved [9] which indicates that environmental conditions influence stress response. Understanding the complexity of the stress-related gene regulation networks, and the adaptation of LAB strains to harsh environmental conditions, is important for the improvement of the industrial strategies using LAB strains and HHP. 

In LAB, repressor HrcA regulates the expression of class I elements by binding specifically to the CIRCE. CIRCE is involved in the controlling inverted repeat for chaperon expression and was found in the promoter regions of the *groE* and *dnaK* operons, which encode two chaperon complexes GroES-GroEL and HrcA-DnaK-GrpE-DnaJ [8,12,13]. The *hrcA* is located in the dnaK operon and its expression is under autorepression control. In some LAB, the CIRCE sequences were found to be upstreaming the *hrcA-grpE-dnaK* [8], and the mRNA analysis showed rapid induction levels after heat shock [8,9]. Studies on the *dnaK* mutants suggested that induction by the stress factor (low pH) can be mediated by heat-shock regulators, HrcA and CtsR [8] and in *L. paracasei dnaK* mutant the stress sensitivities correlated with the upregulation of the gene [10].

In LAB genes from class III (which are regulated by CtsR repressor) are less conservative. It is suggested that CtsR is a widespread transcriptional repressor involved in heat-shock regulation [2] and in Gram (+) bacteria. It is considered to be a master regulator of the cellular protein quality and controls the genes expression involved in proteostasis. In microorganisms, the stress response is a combination of different transcriptional mechanisms that overlap and lead to complex regulatory networks [14,15]. In this context, a vital role is played by cross-regulation between CtsR and HrcA regulons and it is important in the cross-protection mechanisms in LAB that are exposed to harsh environmental factors. Vogel et al. [16] reported the lack of the alternative sigma factor in LAB may allow the bacteria to adapt to a variety of stresses in different environments.

This study aimed to investigate the effect of the HHP (300 MPa/5 min ) on *dnaK*, *hrcA*, and *ctsR* expression levels in 15 *lactobacilli* strains isolated from food samples. 

## 2. Materials and Methods

### 2.1. LAB Strains and Culture Conditions

The 15 strains of LAB were isolated from food (juices, beverages, and beers) samples obtained commercially, to determine the source of microbial contamination of the products. The strains selected for examination were characterized by phenotypical and phylogenetical characteristics to confirm their identification, as described in Bucka-Kolendo et al. [17]. LAB strains used in the studies as well as the introduced new nomenclature proposed by Zheng et al. [18] are presented in Table 1.

Isolation of LAB strain was performed according to the ISO 15214:2000 method using MRS agar (*Lactobacillus* Agar according to DeMan, Rogosa, and Sharpe, Merck KGaA, Darmstadt, Germany) and incubation at 30 °C for 72 h. Additionally, one strain (DSM 6235) isolated from spoiled beer and collected from the Leibniz Institute—DSMZ German Collection of Microorganisms and Cell Cultures (Braunschweig, Germany) was used. The strains were cultured in MRS broth medium at 30 °C for 48–72 h under anaerobic conditions. The growth kinetics of the strains was assessed in the early stationary phase by optical density measurements OD_600_ (Densitometer DEN-1B, BioSan, Riga, Latvia).

### 2.2. High Hydrostatic Pressure Application

Bacterial cultures from the stationary growth phase were exposed to high-pressure treatment using U 4000/65 apparatus (Unipress, Warsaw, Poland). The volume of the treatment chamber was 0.95 L with a maximum working pressure of 600 MPa. A mixture of distilled water and polypropylene glycol (1:1, *v/v*) was used as the pressure-transmitting fluid. The working temperature of the apparatus was in the range between −10 °C and 80 °C. The chosen application of the 300 MPa for 5 min was based on the previous research (data not shown), to guarantee the induction of the genes under the stress condition, and at the same time to avoid the inactivation of the bacterial cells. 

Pressure up to 300 MPa was generated in 70–80 s, and the release time was 2–4 s. Four milliliters of each culture were subjected to HHP at 300 MPa at an ambient temperature (20 °C) and held for approximately 5 min. The pressurization times reported did not include the come-up and come-down times. For each tested strain, the assays were performed in two independent processes. After the treatment, the samples were frozen on dry ice for further analysis. Unpressurized samples were used as a control.

### 2.3. Plate Count Analytical Methods

The viability of each strain was assayed by counting colony-forming units immediately after HHP processing. Thereafter, ten-fold serial dilutions in the Tryptone Salt broth (Biokar Diagnostics, Beauvais, France) were prepared, and appropriate dilutions of bacteria were spread on MRS agar and incubated at 30 °C for 48–72 h under anaerobic conditions. The plates containing less than 300 CFU/mL were selected for counting. The difference between the number of control and treated bacteria was used to estimate the number of HHP survivors. 

### 2.4. Bacterial RNA Extraction

Total mRNA was extracted from control and pressurized cultures with the use of the RNeasy Protect Bacteria Mini Kit (Qiagen, Hilden, Germany) according to the manufacturer’s protocol. The purity and concentration of the RNA were determined by measuring the absorbance at 260 nm and the 260/280 nm ratio using UV-Vis NanoDrop spectrophotometer (ThermoFisher Scientific, Waltham, MA, USA). The RNA integrity was evaluated on the Qubit 4 Fluorometer (ThermoFisher Scientific, Waltham, MA, USA) with Invitrogen Qubit RNA IQ Assay. Moreover, the quality of the RNA was checked by electrophoresis on 1% agarose gels to reveal any contaminating genomic DNA. The RNA samples were stored at −80 °C.

### 2.5. Real-Time PCR (RT-qPCR) 

Custom TaqMan gene expression assays for *dnaK* (no. AP47ZY7), *hrcA* (no. APXGWAE), and *ctsR* (no. APZTJE9) were used for gene expression analysis. Primer and probe sequences are shown in Table 2. These were synthesised by ThermoFisher Scientific (TFS) and included both customised primer and probe sets, designed via the Applied Biosystems Primer Express™ 2.0 software as well as pre-designed, gene-specific TaqMan^®^ probe and primer sets (TaqMan^®^ Gene Expression Assays, ThermoFisher Scientific, Waltham, MA, USA). The relative expression level of stress-related genes was normalized to an endogenous control *16S* rRNA gene (TagMan assay no. Ba04230899_s1; ThermoFisher Scientific, Waltham, MA, USA ). Stability of *16S rRNA* as endogenous control was measured for control end HPP treatment strains using ΔCT algorithm [19]. Relative expression ratios for target genes were calculated using the 2^−ΔΔCT^ method [20]. 

RT-qPCR was performed in triplicate using a TaqMan™ RNA-to-CT™1-Step Kit (ThermoFisher Scientific, Waltham, MA, USA) and a QuantStudio™ 3 Flex Real-Time PCR System (ThermoFisher Scientific, Waltham, MA, USA). Relative gene expression of *dnaK, hrcA*, and *ctsR* was performed using the QuantStudio Design & Analysis Desktop Software v.1.5.1 (ThermoFisher Scientific, Waltham, MA, USA). 

### 2.6. Statistical Data Analysis

The differences in the relative *dnaK, hrcA,* and *ctsR* expression level between control and HHP treated were determined by Student’s *t*-test and analyzed with the use of GraphPad’s Prism v.7 (GraphPad Software, Inc., San Diego, CA, USA), and TIBCO Statistica data analysis system v.13 (TIBCO Software Inc, Palo Alto, CA, USA; 2017). Protein–protein interaction (PPI) analyses of Lactobacillus dnaK, ctsR, and hrcA protein cluster were performed with the STRING database (string-db.org accessed on 15 September 2021) to evaluate known and predicted protein relationships. 

## 3. Results

### 3.1. The Effect of the HHP on the Bacterial Cells

**Figure 1:**
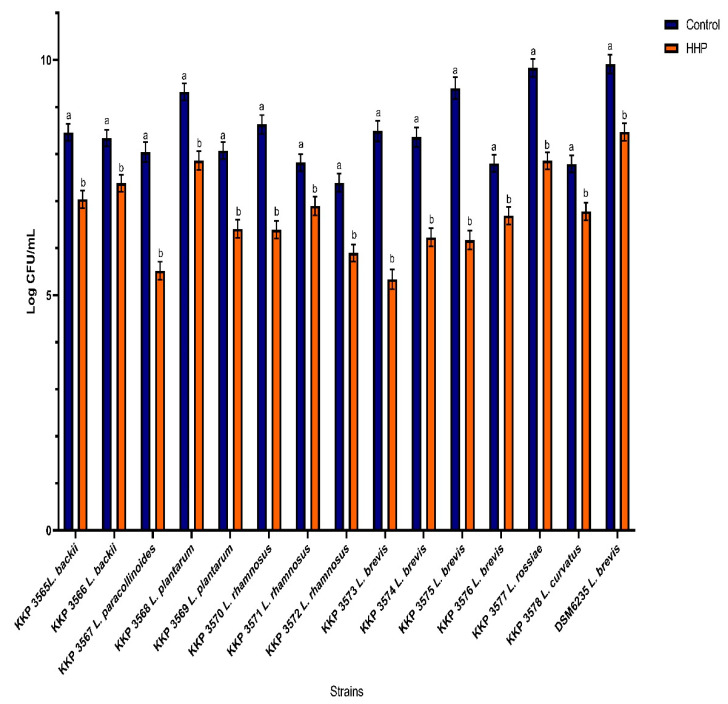
Effect of high hydrostatic pressure on the survival of LAB strains. Presented data are the mean of the two independent experiments and the standard deviations are indicated with vertical bars, (**a**) control strain (**b**) after HHP strain. The bars with different letters are significantly different (*p* < 0.05).

The results of the experiment showed that the LAB subjected to the HHP survived the process, and the survival rates are shown in Figure 1. The pressure of 300 MPa/ 5 min had a significant effect on the survival of the cells (*p* < 0.05) between control and pressurized cells. The highest reduction observed in *L. brevis* KKP 3573 and KKP 3575 was 3.1, and 3.2 logs (CFU/mL) respectively. For strains, *L. paracollinoides* KKP 3567, *L. rhamnosus* KKP 3570, *L. brevis* KKP 3574, the level of reduction was over 2 log (CFU/mL), and for other strains decrease was around 1 log (CFU/mL). 

### 3.2. DnaK Gene 

The relative *dnaK*, *ctsR,* and *hrcA* expression level was investigated in control and HPP treated lactobacilli strains using the RT-qPCR method. Results showed differential patterns of the expression for all three studied genes (*ctsR*, *hrcA*, and *dnaK*). 

**Figure 2:**
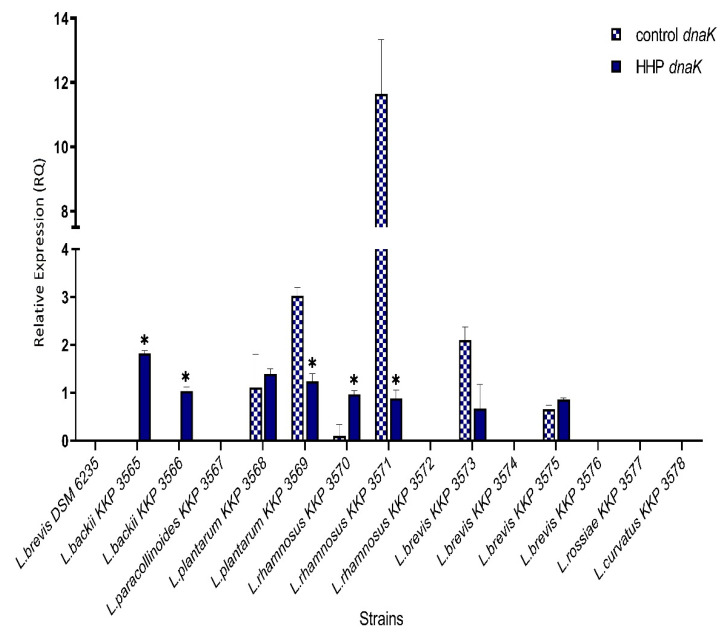
Relative gene expression of *dnaK* in LAB strains before and after exposure to 300 MPa pressure for 5 min. determined by RT-qPCR analysis. Presented data are the mean of the two independent experiments and the standard deviations are indicated with vertical bars. * Statistically significant differences were determined by Student’s *t*-test (*p* < 0.05).

An increase of the *dnaK* expression level in six strains (*L. backii* KKP 3565, *L. backii* KKP 3566, *L. paracollinoides* KKP 3567, *L. plantarum* KKP 3568, *L. rhamnosus* KKP 3570, *L. brevis* KKP 3575) and decrease in three strains (*L. plantarum* KKP 3569, *L. rhamnosus* KKP 3571, *L. brevis* KKP 3573) in comparison to control strains were noticed. The relative expression level of the *dnaK* in *L. brevis* DSM 6235, *L. paracollinoides* KKP 3567, *L. rhamnosus* KKP 3572, *L. brevis* KKP 3574, *L. brevis* KKP 3576, *L. rossiae* KKP 3577, *L. curvatus* KKP 3578 was found to be below to detected limit. Moreover, for five strains, *L. backii* KKP 3565, *L. backii* KKP 3566, *L. plantarum* KKP 3569, *L. rhamnosus* KKP 3570, *L. rhamnosus* KKP 3571, the *dnaK* expression level was statistically significant (*p* < 0.05) between the control and HHP treated groups (Figure 2).

### 3.3. ctsR Gene

**Figure 3:**
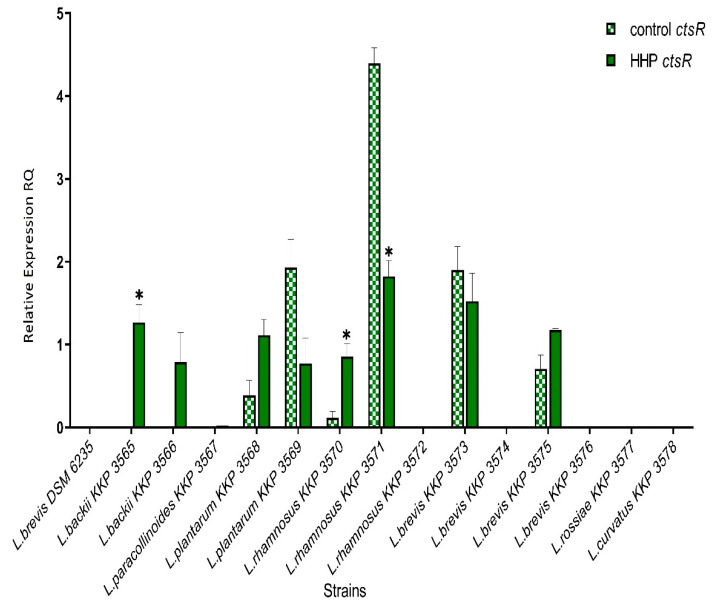
Relative gene expression of *ctsR* in LAB strains before and after exposure to 300 MPa pressure for 5 min. determined by RT-qPCR analysis. Presented data are the mean of the two independent experiments and the standard deviations are indicated with vertical bars. * Statistically significant differences were determined by Student’s *t*-test (*p* < 0.05).

For the *ctsR* gene, an increase of expression for five (*L. backii* KKP 3565, *L. backii* KKP 3566, *L. plantarum* KKP 3568, *L. rhamnosus* KKP 3570, *L. brevis* KKP 3575) strains and a decrease for the three strains (*L. plantarum* KKP 3569, *L. rhamnosus* KKP 3571, *L. brevis* KKP 3573) was noticed. Furthermore, for three strains, *L. backii* KKP 3565 and *L. rhamnosus* KKP 3570, *L. rhamnosus* KKP 3571, *statistically significant differences* (*p* < 0.05) in the level of the *ctsR* expression between control and HHP treated was observed. However, for seven strains (*L. brevis* DSM 6235, *L. paracollinoides* KKP 3567, *L. rhamnosus* KKP 3572, *L. brevis* KKP 3574, *L. brevis* KKP 3576, *L. rossiae* KKP 3577, *L. curvatus* KKP 3578) the level of *ctsR* expression was found both before and after the HHP treatment, under detection limit (Figure 3). 

### 3.4. hrcA Gene

**Figure 4:**
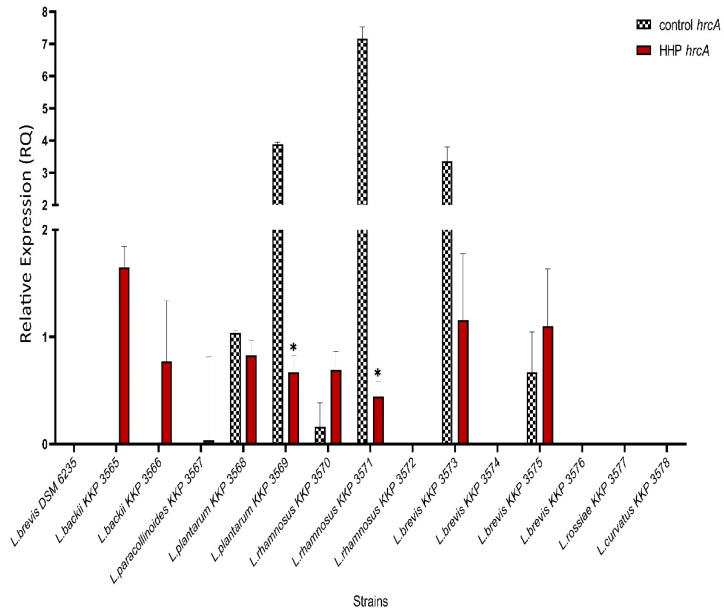
Relative gene expression of *hrcA* in LAB strains before and after exposure to 300 MPa pressure for 5 min determined by RT-qPCR analysis. Presented data are the mean of the two independent experiments and the standard deviations are indicated with vertical bars. * Statistically significant differences were determined by Student’s *t*-test (*p* < 0.05).

For *hrcA* increase of the expression was observed in *L. backii* KKP 3565, *L. backii* KKP 3566, *L. paracollinoides* KKP 3567, *L. rhamnosus* KKP 3570, and *L. brevis* KKP 3575 strains, decrease in four strains (*L. plantarum* KKP 3568, *L. plantarum* KKP 3569, *L. rhamnosus* KKP 3571, *L. brevis* KKP 3573), and for the seven strains (*L. brevis* DSM 6335, *L. paracollinoides* KKP 3567, *L. rhamnosus* KKP 3572, *L. brevis* KKP 3574, *L. brevis* KKP 3576, *L. rossiae* KKP 3577, *L. curvatus* KKP 3578) no expression was observed. For *L. plantarum* KKP 3569 and *L. rhamnosus* KKP 3571 the differences in the *hrcA* expression between control and HHP treated groups were statistically significant (*p* < 0.05) (Figure 4).

Overall, the analysis of the viability showed that the pressurization did affect the studied strains, and the level of survival has decreased compared to the control. The premise of the experiment demonstrates that the conditions of 300 MPa /5′ allows keeping the strains viable but induces changes in the transcriptome and proteome levels. The expression results showed that in six out of 15 strains of *lactobacilli* (*L. brevis* DSM 6235, *L. rhamnosus* KKP 3572, *L. brevis* KKP 3574, *L. brevis* KKP 3576, *L. rossiae* KKP 3577, *L. curvatus* KKP 3578), the level of the *ctsR*, *hrcA*, and *dnaK* expression was not detected either before and after the HHP treatment. In four strains namely, *L. backii* KKP 3565, *L. backii* KKP 3566, *L. rhamnosus* KKP 3570, *L. brevis* KKP 3575, the increase of *ctsR*, *hrcA*, and *dnaK* expression was noticed, whereas, for three strains (*L. plantarum* KKP 3569, *L. rhamnosus* KKP 3571, *L. brevis* KKP 3573) a decrease of *ctsR*, *hrcA* and *dnaK* expression under HHP conditions was observed. For *L. plantarum* KKP 3568 an increase of the *hrcA* expression and a decrease of the *dnaK* and *ctsR* expression was observed. For *L. backii* KKP 3565 and *L. backii* KKP 3566 strains an increase of *ctsR*, *hrcA* and *dnaK* expression levels (1 to 2-fold) after pressurization was determined. However, for *L. rhamnosus* KKP 3571, a significant decrease of *dnaK* (11-fold), *hrcA* (6-fold), and *ctsR* (2,5-fold) expression in response to HHP was observed. In the strain, *L. paracollinoides* KKP 3567, the expression levels for the *dnaK* and *ctsR* were below the detection limit, but a slight increase of the expression *hrcA* after HHP treatment was noticed. The impact of HHP on proteome was previously described in Bucka-Kolendo et al. [17].

## 3.5. Protein–Protein Interaction (PPI) Network Analysis

**Figure 5:**
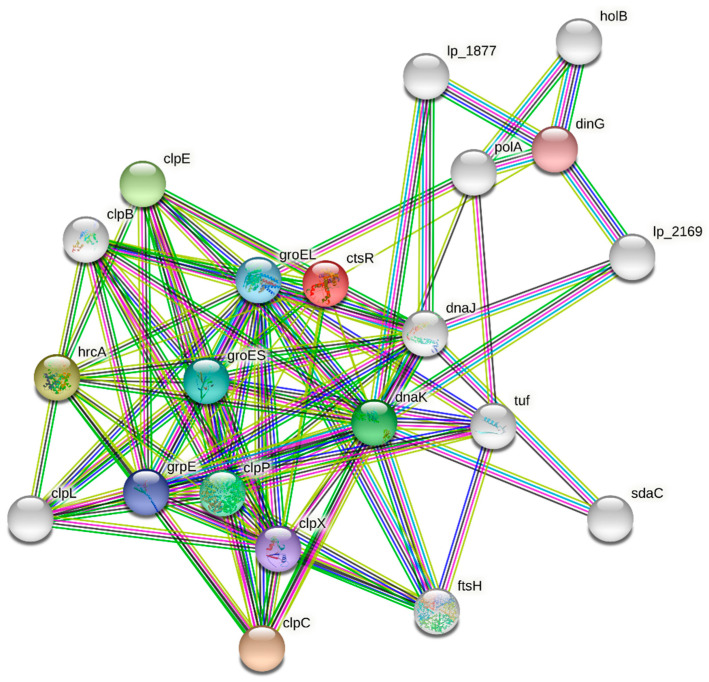
STRING protein–protein interaction (PPI) analyses. PPI network connectivity of Lactobacillus dnaK, ctsR, and hrcA protein cluster. Network contains 98 edges (vs. 27 expected), and enrichment *p*-value < 0.001. The confidence score threshold was set at 0.7 (high) for analyses. The gene interaction data used to build the network was based on direct physical interactions that are either experimentally derived or computationally predicted.

The Search Tool for the Retrieval of Interacting Genes—STRING database (string-db.org) [21] was used to seek potential interactions between differentially expressed *dnaK, ctsR,* and *hrcA* molecules, corresponding to different LAB. PPI network was constructed, with the active interaction sources, including experiments, databases, textriming, co-expression, gene fusion, and co-occurrence. In the matrix, the nodes correspond to the proteins, and the edges represent the interactions. Using the interaction score threshold of 0.7 (high confidence) the string PPI analysis (Figure 5) yielded a highly clustered network (clustering coefficient: 0.73) containing 21 nodes with 98 edges (expected number of edges 27), showing significantly more interactions than expected for a random set of similarity size drawn from the genome (enrichment *p*-value < 0.001). Gene ontology (GO) enrichment analysis of the molecular function included: *protein folding, unfolded protein binding, protein binding* with FDR (false discovery rate) = 1.42 × 10^−0.5^.

The PPI analysis was used to explore future relationships, to establish pathways of protein relationships associated with dnaK, ctsR, and hrcA molecules, that may meet other responses pathways to achieve the most desirable survival phenotype. As the PPI may not be limited to the strain type or physiological conditions, like HHP, the specificity and strength may vary as well. Protein can interact specifically, as when a transcription factor regulates the expression and production of different proteins.

## 4. Discussion

Stress as a result of exposure to different factors can be experienced by LAB in the food industry, particularly during the manufacturing process [1]. Most studies are focused on the ability to cope with heat, cold, osmotic, acid, or oxidative stress, but there is a limited number of studies regarding molecular mechanisms of the stress response, especially under HPP treatment [1,10,17]. While the HHP stress is not common to LAB, cross-protection to other stress factors occurs that induces the response. Vogel et al. [16] showed that reduction of ribosome function may occupy a central position in response to HHP, and can be linked with the expression of stress-related genes. Molecular analysis of the stress-related genes allows the development of tools to screen for tolerant or sensitive strains and to evaluate the adaptation ability of strains to harsh conditions [8]. In addition, for strains grown under different stress conditions, the analysis of transcriptome profiles allowed the identification of gene stress regulatory networks and regulons [11]. Single mutations of the *hrcA* and *ctsR* genes in *L. plantarum* WCFS1 resulted in significant changes in the expression of different genes associated with transcription regulation, primary metabolism, transportation, binding, and biosynthesis of different compounds. The deletion of both *hrcA* and *ctsR* demonstrated the complexity of the cross-talk between the gene regulatory networks influenced by stress-related regulons [11,14]. 

Herein, the relative expression level of the *dnaK*, *ctsR*, and *hrcA* before and after HHP (300 MPa/5′) treatment has been evaluated. Our results showed that LAB under the HHP had different stress toleration patterns. Regulatory mechanisms of stress response genes have been investigated in several Gram-positive bacteria and studied in detail in the genetic model *Bacillus subtilis* [10,22]. Conversely, very little is known about the transcriptional regulation of stress-related genes in *lactobacilli* strains, which are likely to have evolved multiple and complex adaptive mechanisms [8,23]. Moreover, there are not enough reports regarding the impact of HHP treatment on the expression level of these genes of the *lactobacilli* strains, especially isolated from food products. In our study, we observed 1-2 fold higher *hrcA*, *ctsR*, and *dnaK* expression in the pressurized *L. backii* KKP 3565 and *L. backii* KKP 3566 strains whereas a significant decline in the expression levels of the *dnaK* (11-fold, and 1,5-fold), *hrcA* (6-fold, and 3-fold), and *ctsR* (2,5-fold and 1-fold) were noticed for pressurized strains *L. rhamnosus* KKP 3571, and *L. plantarum* KKP 3569, respectively. It has been reported that the *ctsR* mutant *L. plantarum* strain was more resistant to oxidative stress and more sensitive to ethanol and heat stresses than its wild strain, which most likely is related to the pleiotropic character of the gene transcription [11,14]. A possible explanation of these results might be associated with the complexity of the stress-related mechanisms in LAB strains [24], which we also visualized in PPI network analysis. Many transcriptional regulators, such as HrcA, CtsR, Fur, MarR, and MeR family are differentially expressed in response to the heat shock [25,26]. 

It is known, that external signals can elicit dramatic changes in the expression pattern of a variety of stress-related genes encoding proteins thought to improve adaptation to the changing environment [25,26,27,28]. It is thought that under the HHP stress the relative amount of mRNA of many genes is changing, and that can be a result of the selective transcription or mRNA stability [16]. The possible interpretation of the LAB response to HHP may be obtained rather from the transcriptome than from the proteome, as the proteome analysis does not always include low abundance regulatory proteins [10,16]. 

## 5. Conclusions

To summarize, the HHP parameters have to be carefully selected in function during the process in the context of the stress adaptation and identified stress response genes. In this scenario, the following transcriptomic and proteomic studies are needed to understand the impact of the HHP application on the LAB population and to help obtain the knowledge on the LAB with higher behavior flexibility upon stress conditions. Furthermore, clarifying the mechanisms based on the molecular HHP process in LAB can help to control bacterial responses to achieve the desirable robustness of bacteria in relation to various industrial processes. 

Our presented data are the solid base for the understanding of the mechanisms involved in bacterial response during industrial processes and may help to optimize production. Furthermore, the expression level of the targeted genes can point to the intensity of the stress during food and beverage preservation processes and elucidate the role of the *dnaK*, *ctsR*, and *hrcA* genes in the stress-related mechanism. 

## Figures and Tables

**Table 1:** The list of studied lactic acid bacteria with the additional new nomenclature.

Strain	Source	16S rDNA Identification	New Nomenclature
KKP 3565	Beer	*Lactobacillus backii*	*Loigolactobacillus backii*
KKP 3566	Beer	*Lactobacillus backii*	*Loigolactobacillus backii*
KKP 3567	Beer	*Lactobacillus paracollinoides*	*Secundilactobacillus paracollinoides*
KKP 3568	Bread	*Lactobacillus plantarum*	*Lactiplantibacillus plantarum*
KKP 3569	Tomato juice	*Lactobacillus plantarum*	*Lactiplantibacillus plantarum*
KKP 3570	Tomato juice	*Lactobacillus rhamnosus*	*Lacticaseibacillus rhamnosus*
KKP 3571	Probiotic	*Lactobacillus rhamnosus*	*Lacticaseibacillus rhamnosus*
KKP 3572	Ice Cream	*Lactobacillus rhamnosus*	*Lacticaseibacillus rhamnosus*
KKP 3573	Beer	*Lactobacillus brevis*	*Levitlactobacillus brevis*
KKP 3574	Beer	*Lactobacillus brevis*	*Levitlactobacillus brevis*
KKP 3575	Beer	*Lactobacillus brevis*	*Levitlactobacillus brevis*
KKP 3576	Beer	*Lactobacillus brevis*	*Levitlactobacillus brevis*
KKP 3577	Beer	*Lactobacillus rossiae*	*Furfurilactobacillus rossiae*
KKP 3578	Sauerkraut juice	*Lactobacillus curvatus*	*Latilactobacillus curvatus*
DSM6235	Beer	*Lactobacillus brevis*	*Levitlactobacillus brevis*

**Table 2:** Sequence information for primers and probes used.

Gene	Sequence—5′ to 3′	Amplicon (bp)	TagMan Assay No.	GenBank
*ctsR*	F: TGGTCGATGATGCTGATGTG	128	AP47ZY7	CP052869.1
	FAM-ACAACGAGATGCTTATGCGGTCGT-MGB			
	R: TAAATCGTCGAGAAGCGCAA			
*hrcA*	F: TGCAAGATCCAGACGGATTC	116	APXGWAE	JX967738.1
	P: FAM-TTTGGCAGTGTGTTGTCCAAGGC-MGB			
	R: ACTATACGGACCAGCAAAGC			
*dnaK*	F: TGTCGGTCTTATCCAAACC	110	APZTJE9	CP053571.1
	P: FAM-CTGCCGCCGTTGGTTCGTTAATAA-MGB			
	R: CTTTAGTTGCTTGCCGTTGG			
*16S*	Assay by design TFS		Ba04230899_s1	

F—forward; P—probe; R—reverse.
